# Anti-Inflammatory Effects of Cumin Essential Oil by Blocking JNK, ERK, and NF-*κ*B Signaling Pathways in LPS-Stimulated RAW 264.7 Cells

**DOI:** 10.1155/2015/474509

**Published:** 2015-09-06

**Authors:** Juan Wei, Xitong Zhang, Yang Bi, Ruidong Miao, Zhong Zhang, Hailan Su

**Affiliations:** ^1^College of Food Science and Engineering, Gansu Agricultural University, Lanzhou 730070, China; ^2^School of Life Sciences, Lanzhou University, Lanzhou 730000, China

## Abstract

Cumin seeds (*Cuminum cyminum* L.) have been commonly used in food flavoring and perfumery. In this study, cumin essential oil (CuEO) extracted from seeds was employed to investigate the anti-inflammatory effects in lipopolysaccharide- (LPS-) stimulated RAW 264.7 cells and the underlying mechanisms. A total of 26 volatile constituents were identified in CuEO by GC-MS, and the most abundant constituent was cuminaldehyde (48.773%). Mitochondrial-respiration-dependent 3-(4,5-dimethylthiazol-2-yl)-2,5-diphenyltetrazolium (MTT) reduction assay demonstrated that CuEO did not exhibit any cytotoxic effect at the employed concentrations (0.0005–0.01%). Real-time PCR tests showed that CuEO significantly inhibited the mRNA expressions of inducible nitric oxide synthase (iNOS), cyclooxygenase (COX-2), interleukin- (IL-) 1, and IL-6. Moreover, western blotting analysis revealed that CuEO blocked LPS-induced transcriptional activation of nuclear factor-kappa B (NF-*κ*B) and inhibited the phosphorylation of extracellular signal regulated kinase (ERK) and c-Jun N-terminal kinase (JNK). These results suggested that CuEO exerted anti-inflammatory effects in LPS-stimulated RAW 264.7 cells via inhibition of NF-*κ*B and mitogen-activated protein kinases ERK and JNK signaling; the chemical could be used as a source of anti-inflammatory agents as well as dietary complement for health promotion.

## 1. Introduction

Inflammation is a normal protective response induced by tissue injury or infection to combat invaders in the body (microorganisms and non-self cells) and to remove dead or damaged host cells. However, chronic and extreme inflammations cause many inflammatory diseases, such as cancers, rheumatoid arthritis, atherosclerosis, periodontitis, and chronic hepatitis [1, 2]. It has been generally accepted that the excessive production of proinflammatory cytokines and mediators, such as interleukin-1 (IL-1), IL-6, tumor necrosis factor-*α* (TNF-*α*), nitric oxide (NO), and prostaglandin E_2_ (PGE_2_), plays an important role in the development of these inflammatory disease [3]. Therefore, development of potential therapeutic approaches that help to reduce the expression of these proinflammatory genes would be useful in the treatment of many chronic diseases with an underlying inflammatory origin.

Essential oils are complex mixture isolated from aromatic plants. Some essential oils have anti-inflammatory effects [4, 5]. Kim et al. [6] reported the anti-inflammatory effect of fingered citron (*Citrus medica *L. var.* sarcodactylis*) essential oil. Woguem et al. [7] demonstrated that African pepper (*Xylopia parviflora*, Annonaceae) essential oil possessed notable anti-inflammatory potential. Cumin (*C. cyminum *L.) is a spice plant belonging to the family Umbelliferae; its seed has been commonly used in food flavoring and perfumery. Cumin seeds contain nearly 3-4% of essential oil [8]. The cumin essential oil (CuEO) has attracted great attention during the last few years due to the large variety of biological activities that it exhibits, such as antiallergic, antioxidant, antimicrobial, antiplatelet aggregation, and hypoglycemic activities [9–12]. However, there is no report available on anti-inflammatory activity of CuEO.

The objective of this work was therefore to evaluate the anti-inflammatory effects of CuEO, to determine its ability to inhibit the activation of nuclear factor-kappa B (NF-*κ*B) and mitogen-activated protein kinases (MAPKs) in lipopolysaccharide- (LPS-) stimulated RAW 264.7 cells and to clarify its possible mechanism of anti-inflammatory effect.

## 2. Materials and Methods

### 2.1. Plant Material

Cumin (*C. cyminum *L. cv Dunyu number 1) was harvested in Yumen City of Gansu Province, China, in July 2013. Seeds were dried, cleaned, packed and transported to the lab, and then ground by a small grinder.

### 2.2. Reagents

Trizol, Dulbecco's modified Eagle's medium (DMEM), and other cell-culture reagents including fetal bovine serum (FBS) were obtained from Invitrogen-Gibco (Grand Island, NY, USA). Dimethyl sulfoxide (DMSO), phosphate buffered saline (PBS),* Lipopolysaccharide*,* Escherichia coli* 0127:138 (LPS), and 3-(4,5-dimethylthiazol-2yl)-2,5-diphenyltetrazolium bromide (MTT) were purchased from Sigma-Aldrich (St. Louis, MO, USA). Anti-NF-*κ*B, anti-JNK, anti-phosphorylated JNK, anti-ERK, anti-phosphorylated ERK, anti-p38, and anti-phosphorylated p38 mouse or rabbit antibodies were purchased from BD Biosciences (San Diego, CA, USA). The polymerase chain reaction (PCR) primers of iNOS, COX-2, IL-1*β*, IL-6, and *β*-actin were synthesized by Sangon Biotech Co., Ltd. (Shanghai, China). Reverse transcription and real-time PCR kits were purchased from Bio-Rad Laboratories (Hercules, CA, USA). M-PER Mammalian Protein Extraction Reagent kit and NE-PER Nuclear and Cytoplasmic Extraction kit (Thermo Scientific) were purchased from Thermo Scientific (Waltham, MA, USA).

### 2.3. Essential Oil Extraction

Seeds powder (200 g) was subjected to water distillation in a Clevenger-type apparatus for 3 h according to the method recommended by Singh et al. [13] The yield (v/w) of essential oil was determined based on the dry weight of seeds. The obtained essential oil was fried over anhydrous sodium sulfate, filtered, and stored in a sealed vial at 4°C until tested.

### 2.4. Cell Culture

Followed by the method of Kim et al. [6], the macrophage cell line RAW 264.7 was obtained from American Type Cell Culture (Bioleaf Company, Shanghai, China) and cultured in DMEM containing 10% FBS and 1% penicillin-streptomycin solution (100 U/mL penicillin and 100 ug/mL streptomycin in 0.85% NaCl). The cells were incubated in an atmosphere of 5% CO_2_ at 37°C and were subcultured every 2 or 3 d.

### 2.5. GC-MS Analysis

The compositions of cumin essential oil were analyzed on an Agilent 6890 gas chromatograph (GC) connected to an Agilent 5973 mass spectrometer (MS) using an HP-5MS (5% phenylmethylpolysiloxane, 30 m, 0.25 mm i.d., and 0.1 um film thickness; J & W Scientific) capillary column. The temperature program was 1 min at 80°C, subsequently 4°C/min up to 180°C, and then 8°C/min up to 250°C, held for 5 min; the injector and transfer line temperatures were 270°C. Helium was used as the carrier gas at a flow rate of 1 mL/min, and the split ratio was 1 : 50; acquisition mass range was from 40 to 500* m/z* units. Oil samples were diluted 1 : 100 in *n*-hexane and the volume injected was 2 uL. Quantification was obtained from percentage peak areas from the gas chromatogram. Wiley (V.7.0)/NBS (V.2.0) Registry of Mass Spectral Database libraries search and/or authentic reference compounds and comparison of their Kovats index were used for substance indemnification.

### 2.6. MTT Cytotoxicity Assay

According to the method described by Ko and Jeon [14], the cytotoxicity of CuEO was assessed by mitochondrial-respiration-dependent 3-(4,5-dimethylthiazol-2-yl)-2,5-diphenyltetrazolium (MTT) reduction method. RAW 264.7 cells (1 × 10^4^ cells/well) plated on 96-well plates were treated with different concentrations of CuEO for 24 and 48 h at 37°C in 5% CO_2_. Then, the cells were washed with PBS and incubated with MTT 1 mg/mL in PBS for 4 h at 37°C in 5% CO_2_. Afterwards, the plates were centrifuged for 10 min at 2000 rpm, and the supernatants were aspirated. The formazan crystals in each well were dissolved in DMSO. The amount of purple formazan was assessed by measuring the absorbance at 540 nm using a microplate reader (Sunrise Remote, Tecan). The optical density of formazan formed in control cells (without treatment with CuEO) was taken as 100% viability.

### 2.7. Real-Time PCR of mRNA Inflammatory Cytokines and Related Inflammatory Mediators

#### 2.7.1. Total RNA Isolation

Methods followed Liang et al. [15] with some modifications. Cells were plated onto 6-well plates at a density of 1 × 10^5^ cells/well and incubated with CuEO for 1 h prior to LPS (1 ug/mL) stimulation. After 24 h, cells were lysed and total RNA extraction was performed by using Trizol reagent from Invitrogen. Cells were homogenized in 200 uL of Trizol reagent, and then samples were left to rest at room temperature for 5 min. After that, 40 uL of chloroform was added and the tubes were vigorously shaken for 15 s and allowed to rest at room temperature for 5 min. Tubes were then centrifuged at 12000 g (VWR, Galaxy 4D, diameter 14 cm), 4°C for 15 min. The aqueous phase was transferred to a new tube. Isopropyl alcohol (100 uL) was added to the aqueous phase: the tube was then gently mixed and incubated at room temperature for 10 min. After incubation, samples were centrifuged at 12000 g, 4°C for 10 min. the supernatant was poured and the pellet was washed by 200 uL of 75% ethanol and centrifuged at 7600 g, 4°C for 5 min. The washing step and centrifuge were repeated. The final supernatant was removed and the pellet was dried until being colorless. Total RNA was then dissolved in 20 uL of DEPC H_2_O, incubated at 65°C for 5 minutes, and stored at −80°C until being used.

#### 2.7.2. Gene Expression Quantification

The expression of mRNA transcripts of iNOS (forward: GTTCTCAGCCCAACAATACAAGA, reverse: GTGGACGGGTCGATGTCAC), COX-2 (forward: TTCCAATCCATGTCAAAACCGT, reverse: AGTCCGGGTACAGTCACACTT), IL-1*β* (forward: GAAATGCCACCTTTTGACAGTG, reverse: TGGATGCTCTCATCAGGACAG), IL-6 (forward: CTGCAAGAGACTTCCATCCAG, reverse: AGTGGTATAGACAGGTCTGTTGG), and *β*-actin (forward: GGCTGTATTCCCCTCCATCG, reverse: CCAGTTGGTAACAATGCCATGT) was determined by real-time RT-PCR. cDNA was synthesized from total RNA using oligo(dT) 15 primers. iQ SYBR Green Supermix (Bio-Rad Laboratories, Inc., Hercules, CA, USA) and iCvcler iQ Real-Time PCR Detection System (Bio-Rad Laboratories, Inc.) were used for real-time PCR analysis. Using standards, the amount of iNOS, COX-2, IL-1*β*, and IL-6 cDNA was determined and normalized by the amount of *β*-actin cDNA.

### 2.8. Western Blot Detection of NF-*κ*B and MAPK Activation

The experiment was carried out according to the method described by Kim et al. [16] with modifications. RAW 264.7 cells were seeded in 10 cm cell-culture dishes at a density of 3 × 10^6^ cells per dish and cultured overnight. Cells were then pretreated with CuEO (0.001%, 0.01%) for 1 hour and stimulated with LPS (1 ug/mL) for 30 min. After incubation, the cells were collected and washed twice with cold PBS. Total cellular proteins were extracted by M-PER Mammalian Protein Extraction Reagent kit (Thermo Scientific). Cytoplasmic and nuclear proteins were extracted by using the NE-PER Nuclear and Cytoplasmic Extraction kit (Thermo Scientific). Proteins were then maintained at −80°C for western blot analysis. Protein concentrations in the supernatant fractions were determined using a BCA protein assay (Sigma); then, proteins were applied to sodium dodecyl sulfate-polyacrylamide gel and transferred to polyvinylidene fluoride membranes. After blocking for 60 min in a 5% skim milk solution, membranes were incubated overnight at 4°C with primary antibodies against P-JNK, JNK, p-ERK, ERK, p-p38, p38, and NF-*κ*B p65, followed by horseradish peroxidase-conjugated secondary antibody at room temperature for 1 h. The reactive bands were visualized by enhanced chemiluminescence.

### 2.9. Statistical Analysis

Statistical analysis was performed by the SPSS 17.0 software (SPSS, Inc., Chicago, IL). All data are expressed as the mean ± SD. The differences between groups were analyzed by Student's *t*-test. A value of *p* < 0.05 and *p* < 0.01 was considered statistically significant.

## 3. Results

### 3.1. Chemical Composition of CuEO

**Figure 1:**
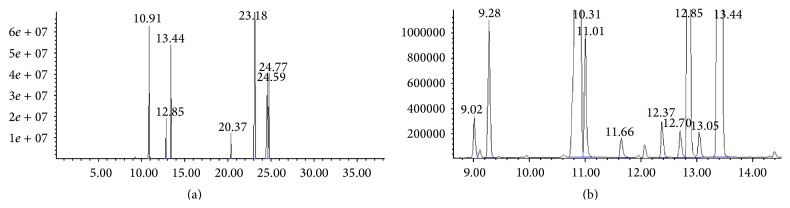
GC-MS total ion chromatogram of CuEO. (a) Retention time was from 0 to 35 min; (b) retention time was from 9 to 14 min. The compound labels in the chromatogram correspond to the Arabic numeral given in Table 1.

As shown in Figure 1 and Table 1, a total of 26 volatile constituents were identified in CuEO, representing 99.81% of the total essential oil on the basis of their mass spectra. The most abundant constituent in essential oil was cuminaldehyde (48.773%), followed by 3-caren-10-al (14.000%) and *β*-pinene (11.438%).

### 3.2. Cytotoxicity of CuEO

**Figure 2:**
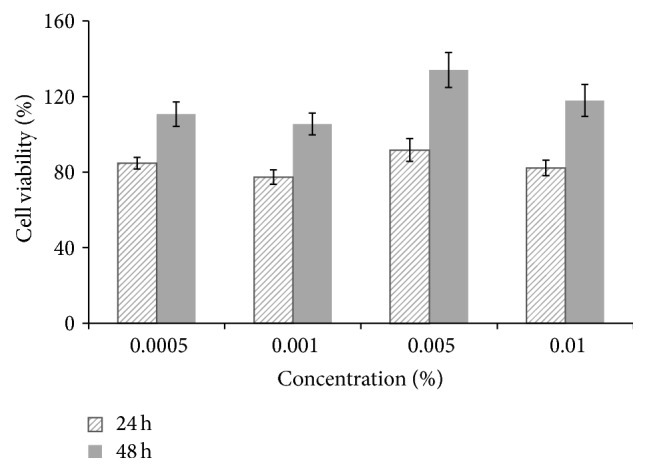
Cytotoxicity of CuEO at different concentrations on the viability of RAW 264.7 cells. Cells (1 × 10^4^ cells/well) plated on 96-well plates were treated with various concentrations of CuEO at 37°C for 24 h. Cytotoxicity of CuEO was assessed by MTT assay. Values are expressed as mean ± SD, *n* = 6.

There was no significant decrease in cell viability for concentrations up to 0.01% after treatment with CuEO for 24 and 48 h (Figure 2). The results indicated that CuEO did not exhibit any cytotoxic effect at the employed concentrations (0.0005–0.01%). Therefore, the concentrations of CuEO ranging from 0.005% to 0.01% were employed for subsequent experiments.

### 3.3. Inhibitory Effect of CuEO on LPS-Induced iNOS and COX-2 mRNA Expression

**Figure 3:**
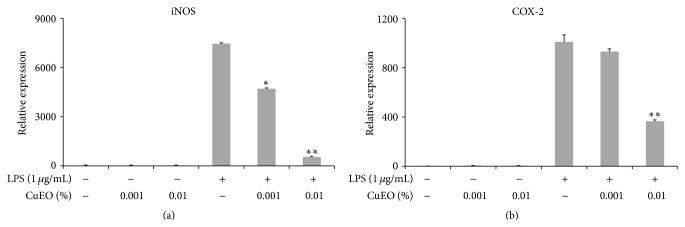
Effect of CuEO at different concentrations on the mRNA expression of LPS-induced iNOS (a) and COX-2 (b) of RAW 264.7 cells. The cells (1 × 10^5^ cells/well in a six-well plate) were pretreated with CuEO (0.001% to 0.01%) for 1 h and then stimulated with LPS (1 ug/mL). After 24 h incubation, total RNA was isolated and the mRNA expression was determined by real-time RT-PCR. Data represent the mean ± SD of three different experiments. ^*∗*^ *p* < 0.05 and ^*∗∗*^ *p* < 0.01 versus LPS-treated alone.

Stimulation of macrophages with LPS resulted in a strong increase in iNOS and COX-2 mRNA expression (Figures 3(a) and 3(b)). A dose-dependent decrease in iNOS and COX-2 mRNA expressions was noted in cells treated with CuEO. The iNOS and COX-2 mRNA levels in cells treated with 0.01% CuEO were 7.2% and 36.1% of those of cells treated with LPS alone. The result indicated that CuEO exerted anti-inflammatory effect.

### 3.4. Inhibitory Effect of CuEO on LPS-Induced IL-1*β* and IL-6 mRNA Expression

**Figure 4:**
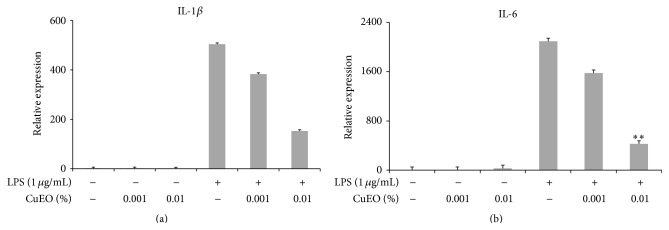
Effect of CuEO at different concentrations on the mRNA expression of LPS-induced IL-1*β* (a) and IL-6 (b) of RAW 264.7 cells. The cells (1 × 10^5^ cells/well in a six-well plate) were pretreated with CuEO (0.001% to 0.01%) for 1 h and then stimulated with LPS (1 ug/mL). After 24 h incubation, total RNA was isolated and the mRNA expression was determined by real-time RT-PCR. Data represent the mean ± SD of three different experiments. ^*∗*^ *p* < 0.05 and ^*∗∗*^ *p* < 0.01 versus LPS-treated alone.

IL-1*β* and IL-6 levels were significantly increased in LPS-activated cells compared to nonactivated controls (Figures 4(a) and 4(b)). Pretreatment of LPS-activated cells with CuEO resulted in an overall reduction of proinflammatory cytokines released in a dose-dependent manner. IL-1*β* and IL-6 mRNA expressions were reduced to 30.2% and 1.3% of LPS alone-treated cells after treatment with 0.01% CuEO. Real-time PCR results demonstrated that CuEO had a better anti-inflammatory effect (Figures 3 and 4).

### 3.5. Effect of CuEO on LPS-Induced Nuclear Translocation of NF-*κ*B

**Figure 5:**
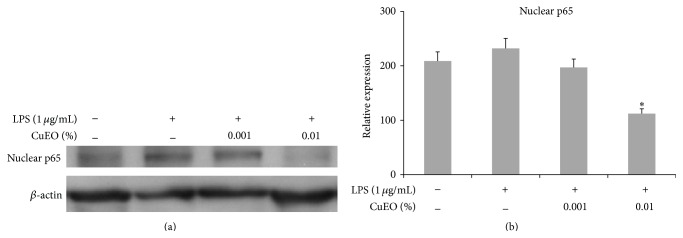
Effect of CuEO on NF-*κ*B p65 nuclear translocation in LPS-stimulated RAW 264.7 cells. (a) The bands of NF-*κ*B p65 proteins tested by western blotting. (b) Data quantification of NF-*κ*B p65. The bands of western blotting were quantified and expressed as the ratio of p65/*β*-actin intensity. The cells (3 × 10^6^ cells per dish) were pretreated with CuEO (0.001% and 0.01%) for 1 h and then stimulated with LPS (1 ug/mL). After 30 min incubation, cells were collected and cytoplasmic and nuclear proteins were extracted. Actin was used as an internal control. Data represent the mean ± SD of three different experiments. ^*∗*^ *p* < 0.05 and ^*∗∗*^ *p* < 0.01 versus LPS-treated alone.

CuEO treatment remarkably reduced the nuclear NF-*κ*B p65 levels in LPS-stimulated RAW 264.7 cells (Figure 5). 0.01% CuEO caused 52% inhibition of nuclear NF-*κ*B p65 levels in LPS alone-treated cells. The results demonstrated that CuEO exhibited anti-inflammatory effect partially by blocking NF-*κ*B activation.

### 3.6. Effect of CuEO on the Phosphorylation of MAPK Signaling Pathways

**Figure 6:**
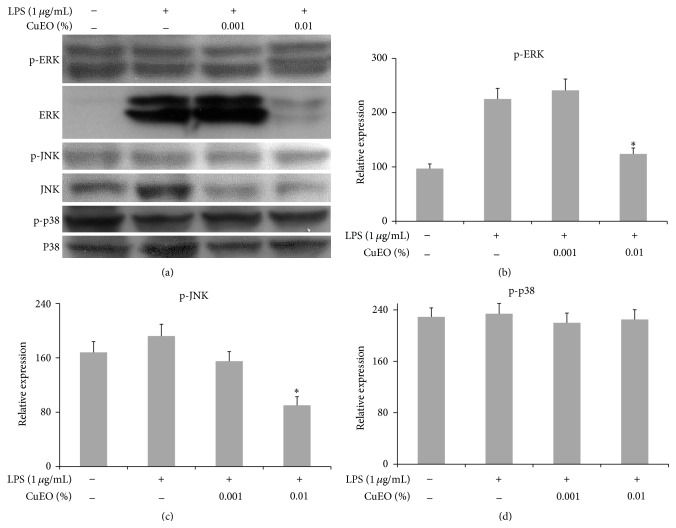
Effect of CuEO on the protein phosphorylation levels of MAPK pathways in LPS-stimulated RAW 264.7 cells. (a) The bands of MAPK proteins tested by western blotting. (b) Data quantification of p-ERK. (c) Data quantification of p-JNK. (d) Data quantification of p-p38. The bands of western blotting were quantified and expressed as the ratio of p-ERK/ERK, p-JNK/JNK, and p-p38/p38 intensity. The cells (3 × 10^6^ cells per dish) were pretreated with CuEO (0.001% and 0.01%) for 1 h and then stimulated with LPS (1 ug/mL). After 30 min incubation, cells were collected and total cellular proteins were extracted. Data represent the mean ± SD of three different experiments. ^*∗*^ *p* < 0.05 and ^*∗∗*^ *p* < 0.01 versus LPS-treated alone.

Pretreatment with CuEO greatly suppressed LPS-induced phosphorylation of JNK and ERK but slightly suppressed p38 phosphorylation (Figure 6). The treatment with 0.01% CuEO resulted in 45% and 53% inhibition of LPS-induced JNK and ERK phosphorylation. These results suggested that the anti-inflammatory effect of CuEO was partially attributed to regulating JNK and ERK pathways.

## 4. Discussion

In this study, we found that CuEO exerted promising anti-inflammatory effects in LPS-stimulated RAW 264.7 cells (Figures 3 and 4). Meanwhile, our results further indicated that CuEO attenuated LPS-induced inflammatory response via NF-*κ*B, JNK, and ERK pathways.

Inflammation is a physiological process that initiates in response to bacterial infection or tissue damage. Macrophages play substantial roles in host defense against infection and can be activated by pathogen- or host-derived molecules, such as lipopolysaccharide (LPS). Once activated, macrophages could secrete excessive proinflammatory enzymes, such as inducible NO synthase (iNOS) and cyclooxygenase (COX-2) [17, 18]. iNOS catalyzes the oxidative deamination of L-arginine and finally produces a large amount of nitric oxide (NO). Continuous production of NO causes harmful effects in the pathogenic cascade of many inflammatory diseases [19]. COX-2 is a key enzyme involved in the biosynthesis of prostaglandin E2 (PGE2) [17]. PGE2 also has been implicated as an important mediator in inflammation [20]. Chang et al. [21] reported that the generation of PGE2 was closely related to NO production. Thus, reducing the levels of iNOS and COX-2 would be an effective strategy for suppressing inflammation. In our study, we found that 0.01% CuEO effectively inhibited iNOS and COX-2 mRNA expression in LPS-stimulated RAW 264.7 cells (Figure 3). Similar reports have been found for other essential oils from oregano (*Origanum vulgare*) and citrus (*Citrus sunki*) [22, 23].

Our present study also demonstrated that 0.01% CuEO significantly decreased mRNA levels of IL-1*β* and IL-6 (Figure 4). It is known that IL-1*β* and IL-6 are important key factors in inflammatory response and are also involved in various chronic inflammatory diseases [24]. Therefore, the inhibition of these cytokines' production or function is a key mechanism in the control of inflammation. It is reported that some essential oils inhibited the expression of some proinflammatory cytokines in the process of macrophage activation. Kim et al. [6] demonstrated that the fingered citron essential oil exerted an anti-inflammatory effect by inhibiting IL-1*β*, IL-6, and TNF-*α* production.

NF-*κ*B is known to play a critical role in the regulation of cell survival genes and to induce the expression of inflammatory enzymes and cytokines, such as iNOS, COX-2, IL-1*β*, and IL-6 [25]. NF-*κ*B p65 is a pivotal member of NF-*κ*B transcription factor family. Under normal physiological conditions, NF-*κ*B binds to I*κ*B inhibitor protein in promoter region via its p65 subunit, which is the inactive form. Once activated by LPS or other external antigens, I*κ*B is phosphorylated by I*κ*B kinases (IKK) leading to proteasome-dependent degradation of I*κ*B, which allows a rapid translocation of NF-*κ*B p65 into the nucleus [26]. Thus, inhibition of NF-*κ*B activation could decrease inflammatory responses. It is reported that some essential oils exert anti-inflammatory activity by suppressing LPS-induced NF-*κ*B activation [6, 27]. In this study, we found that CuEO prevented the nuclear translocation of NF-*κ*B p65 subunit induced by LPS in RAW 264.7 cells (Figure 5). Our results suggest that the suppression of iNOS, COX-2, IL-1*β*, and IL-6 expression by CuEO is partially mediated by inhibition of NF-*κ*B activation. Moreover, these data suggest this occurs at the level of transcription as NF-*κ*B is a transcription factor.

In addition to NF-*κ*B, MAPK pathways including ERK, JNK, and p38 also play a critical role in inflammatory response [28]. Several studies have shown that MAPKs are the upstream enzymes and signaling molecules for NF-*κ*B [29]. We also found that CuEO treatment significantly inhibited the phosphorylation of ERK and JNK, while it had no effect on the phosphorylation of p38 (Figure 6). The results suggest that inhibition of MAPK phosphorylation might be partially involved in anti-inflammatory effects of CuEO in LPS-induced RAW 264.7 cells. In fact, it is well known that the effect of MAPK family in cellular responses depends on the cell type and stimulus conditions [30]. This might be the reason why CuEO blocked the phosphorylation of ERK and JNK in LPS-induced inflammation, but the phosphorylation of p38 was not affected.

In conclusion, the current study clearly demonstrated that CuEO significantly suppressed inflammatory response in LPS-stimulated RAW 264.7 cells via inhibition of NF-*κ*B activation and MAPK ERK and JNK phosphorylation. These results were coincident with a research of the anti-inflammatory effect of standardized aqueous extract of* Cuminum cyminum* seeds in hypertensive rats [31]. As CuEO has little toxicity, it could be used in the development of new functional foods for the prevention or treatment of inflammation-based chronic diseases. Thus, it is worthy of further investigations to clarify the availability and the effectiveness of CuEO* in vivo* and to establish the composition-activity relationship. In our study, a total of 26 volatile constituents were identified in CuEO from Yumen City of Gansu Province, and the most abundant constituent was cuminaldehyde (48.773%), followed by 3-caren-10-al (14.000%) and *β*-pinene (11.438%) (Figure 1 and Table 1). Therefore, the studies on anti-inflammatory effects of those major components are now carried on in our lab.

## Figures and Tables

**Table 1:** Chemical components of cumin essential oil.

Number	Constituent	RT (min)	Peak area (%)
1	*α*-Phellandrene	9.02	0.081
2	*α*-Pinene	9.28	0.159
3	*β*-Pinene	10.91	11.438
4	*α*-Myrcene	11.01	0.146
5	Terpinolene	11.66	0.018
6	Limonene	12.37	0.049
7	*β*-Phellandrene	12.70	0.037
8	Benzene	12.85	2.642
9	Eucalyptol	13.05	0.037
10	*γ*-Terpinene	13.44	10.698
11	Linalool	16.58	0.011
12	2,6-Dimethyl-3,5,7-octatriene-2-ol	19.10	0.011
13	4-Methyl-1-(1-methylethyl)-	19.43	0.031
14	1,3-Cyclohexadiene-1-methanol	20.37	2.023
15	Cuminaldehyde	23.18	48.773
16	3-Cyclohexene-1-carboxaldehyde, 1,3,4-trimethyl-	24.07	0.064
17	1H-Cyclopropa[a]naphthalene,1a,2,3,5,6,7,7a,7b-octahydro-1,1,7,7a-tetramethyl-, [1aR-(1aà,7à,7aà,7bà)]-	24.26	0.034
18	3-Caren-10-al	24.59	14.000
19	2-Caren-10-al	24.77	9.355
20	Benzenemethanol, 4-(1-methylethyl)-	25.35	0.078
21	Bicyclo[3.1.1]hept-2-ene-2-methanol, 6,6-dimethyl-	25.85	0.016
22	cis-à-Bisabolene	26.29	0.026
23	1,6,10-Dodecatriene, 7,11-dimethyl-3-methylene-, (E)-	26.52	0.029
24	*γ*-Elemene	27.70	0.031
25	Naphthalene	27.78	0.012
26	Carotol	31.81	0.011
	Total identified		99.81

RT: retention time.
